# *In vitro* and clinical studies examining the expression of osteopontin in cigarette smoke-exposed endothelial cells and cigarette smokers

**DOI:** 10.1186/1471-2261-12-75

**Published:** 2012-09-17

**Authors:** Emma Bishop, Eugenia H Theophilus, Ian M Fearon

**Affiliations:** 1 British American Tobacco, Group Research and Development, Regents Park Road, Southampton SO15 8TL, UK; 2 R.J. Reynolds Tobacco Company, Winston-Salem, NC 27105, USA

**Keywords:** Osteopontin, *In vitro*, Endothelial cells, MMP-3, Smoking, Atherosclerosis

## Abstract

**Background:**

Cigarette smoking is a leading cause of mortality and morbidity and is associated with cardiovascular disease via contributory processes such as endothelial dysfunction, inflammation and thrombosis. Cigarette smoke both contains and stimulates the production of cellular oxidants and it may also promote vascular inflammation. Osteopontin is a non-collagenous matrix protein first identified in bone and there is increasing evidence for its role in inflammation and cardiovascular disease via its action as a soluble cytokine.

**Methods:**

In this study we have examined the mechanisms underlying the expression of osteopontin in human vascular endothelial cells *in vitro* following exposure to cigarette smoke particulate matter (PM), using PCR, electrochemiluminescence, immunostaining and Western blotting. We further determined if serum osteopontin levels changed in humans who quit smoking.

**Results:**

Non-cytotoxic concentrations of PM increased osteopontin levels in cultured human endothelial cells and this effect was reduced in the presence of ascorbate, suggesting a role for oxidants in the response to PM. However, oxidant production played no role in the PM-evoked induction MMP-3, an enzyme which cleaves osteopontin. In smokers who quit smoking for 5 days, serum osteopontin levels were significantly lowered compared to those measured prior to smoking cessation.

**Conclusions:**

*In vitro* cigarette smoke extract exposure induced osteopontin expression in human endothelial cells in an oxidative stress-dependent manner, which may involve MMP-3 cleavage. In humans, serum osteopontin was decreased with short-term smoking cessation. Endothelial-derived osteopontin may contribute to inflammation in smokers, and may also contribute to atherosclerosis and cardiovascular disease-related processes.

## Background

Atherosclerotic cardiovascular disease is a leading cause of mortality and morbidity worldwide [1-3]. Numerous risk factors including genetic predisposition, diet, lifestyle and smoking status affect the likelihood of an individual developing cardiovascular disease, specifically atherosclerosis. Cigarette smoking has a long-standing association with cardiovascular disease and there is a wealth of evidence concerning the effects of smoking on key pathological processes such as vascular injury, vascular dysfunction, inflammation and thrombosis [3]. Cigarette smoke is a complex and dynamic mixture of more than 5,600 identified chemical constituents [4], many of which may interfere with a number of cellular processes and promote atherosclerosis [5]. Presently, a full understanding of the mechanisms underlying the susceptibility of smokers to cardiovascular disease is lacking, as is our knowledge concerning which smoke constituents, either individually or in combination with others, contribute to its pathogenesis. Greater understanding of the mechanisms and chemical constituents involved and biological changes that occur with smoking cessation could aid in the treatment and prevention of smoking-induced cardiovascular disease.

Osteopontin is a non-collagenous matricellular protein [6] which was first identified as a component of bone and is involved in bone formation and calcification [7]. Recent studies have shown an emerging and more widespread role for osteopontin in inflammation, tissue remodelling and vascular disease via its action as an inflammatory cytokine [8-10]. Soluble osteopontin interacts with cell surface integrins to regulate cell adhesion, migration and proliferation. Thrombin and matrix metalloproteinases (MMPs) can differentially cleave osteopontin at the amino acid sequence glycine-arginine-glycine-aspartic acid-serine (termed the GRGDS) motif which can disrupt integrin binding, ultimately altering cellular responses [8]. MMP-regulated osteopontin is also suggested to have a role in tissue injury [11]. Clinical studies have strengthened the association between osteopontin and cardiovascular disease and osteopontin is now thought to represent a biomarker of both the progression and severity of coronary artery disease [12], as well as a therapeutic target for cardiovascular disease interventions [13]. Osteopontin is expressed by macrophages and acts as a potent macrophage-chemotactic stimulus, regulating macrophage infiltration and retention at sites of chronic inflammation [8]. Osteopontin expression has also been detected in angiogenic endothelial cells, smooth muscle cells and macrophages from atherosclerotic plaques, suggestive of a role in lesion remodelling [8].

Given the link between inflammation, atherosclerosis and smoking, and also given the emerging evidence of a role for osteopontin in cardiovascular disease, we have examined the expression of osteopontin in endothelial cells exposed to cigarette smoke *in vitro* and in smokers’ serum. Our findings advance our knowledge of the potential role for this important inflammatory factor in cigarette smoke-induced cardiovascular disease.

## Methods

### Cigarette smoke total particulate matter generation

Particulate matter from cigarette smoke was generated using 3R4F reference cigarettes (University of Kentucky). Cigarettes were conditioned for a minimum of 48 h at 22°C and 60% humidity (ISO 3402:1999) before smoking on a RM20S smoking machine (Borgwaldt KC, Hamburg, Germany) under ISO standard conditions (35 ml puff taken over 2 sec. every minute; ISO 3308:2000). Smoke particulate matter (PM) was collected on a Cambridge filter pad at a minimum of 250 mg tar per pad. These are non-ISO standard conditions designed to provide a higher yield of particulate matter than conventionally achieved. PM was eluted with dimethylsulphoxide (DMSO) at 24 mg/ml and stored protected from light in single use aliquots at -20°C for no longer than 1 month.

### Cell culture and treatment

Human umbilical vein endothelial cells (HUVECs; Lifeline Cell Technology, California, U.S.A.) were maintained at 37°C in a 5% CO_2_ humidified atmosphere. Cells were cultured in VascuLife® VEGF media (Lifeline Cell Technology) supplemented with VEGF (5 ng/ml), EGF (5 ng/ml); basic FGF (5 ng/ml), IGF-1 (15 ng/ml), ascorbic acid (50 μg/ml), L-glutamine (10 mM), hydrocortisone hemisuccinate (1.0 μg/ml), heparin sulphate (0.75 units/ml) and foetal bovine serum (2%). Cells were seeded at 1×10^4^ cells/ml for all assays with the exception of PCR experiments where cells were seeded at 2x10^4^ cells/ml. Cells were treated for 24 h either with PM or with 1% DMSO as a diluent control. To examine the contribution of oxidative stress in regulating osteopontin levels, cells were incubated with the anti-oxidant ascorbate (200 μM) for 5 h prior to PM exposure.

### Assessment of cell viability

Cell viability was measured by neutral red uptake assays. In brief, HUVECs were incubated in 150 μl neutral red solution diluted 1:65 in VascuLife® VEGF media. Cells were incubated for 3 h at 37°C to allow active uptake of the dye into viable cells. Plates were washed twice in PBS before the addition of 150 μl de-stain solution (50% ethanol, 49% distilled water and 1% glacial acetic acid). After 20 min. of agitation, the optical density was determined at 540 nm with a reference filter of 630 nm using a microplate spectrophotometer (Thermo Labsystems, Waltham, MA, U.S.A.). Background readings taken from blank wells were subtracted from wells containing both untreated and treated cells. Neutral red uptake was expressed as a percentage of the level in untreated cells.

### Quantification of osteopontin and MMP-3 gene expression

Gene expression was assessed with the RT^2^ profiler™ PCR array system (SABiosciences) using an atherosclerosis-specific panel. Cells were lysed using TRIzol® reagent and centrifuged with chloroform. The supernatant was added to an equal volume of isopropanol and incubated at -20°C to precipitate RNA. Samples were centrifuged to pellet mRNA followed by an ethanol wash. Genomic DNA was eliminated using the supplied First Strand Kit and mRNA was diluted to 1 μg total mRNA. The components of the RT cocktail were combined in a microfuge tube and added in equal volume to RNA. The resultant mixture was incubated at 42°C for 15 min., after which the reaction was inactivated at 95°C for 5 min. cDNA was diluted in dH_2_0 and combined with the PCR mastermix and 25 μl of solution was added to each well of the array plate. The following thermal cycler protocol was used: 10 min. at 95°C, 40 cycles of 15 sec. at 95°C and 1 min. at 60°C.

### Quantification of osteopontin, IL-6 and MMP-3 protein levels

Osteopontin and MMP-3 levels were evaluated by electrochemiluminescence using the MSD® platform (Meso Scale Discovery, MD, U.S.A.), in supernatants collected from HUVEC cultures or sera from smokers and abstainers. 25 μl of conditioned media or serum and calibrators (0-100 ng/ml osteopontin, 0-2,500 pg/ml IL-6 and 0-100 ng/ml MMP-3) were incubated with agitation on the assay plate (osteopontin singleplex, ultrasensitive pro-inflammatory-4 II (IL-6) MULTI-SPOT® and MMP-3-plex MULTI-SPOT® 96-well small spot plates) for 1.5 h. After this, SULFO-TAG anti-human osteopontin, anti-human IL-6 or anti-human MMP-3 antibodies were added to each well at 1 μg/ml. After a further 1.5-hour incubation period, plates were washed in 0.05% (v/v) PBS-Tween-20 solution followed by the addition of 150 μl Read Buffer. Plates were read using a MSD® SECTOR 2400 imager and protein concentration assessed against a standard curve, using MSD Workbench® software.

### Detection of osteopontin by immunocytochemistry

After PM treatment, HUVECs were washed in PBS and fixed in ice-cold methanol for 15 min. Cells were then blocked in PBS containing 2% BSA for 45 min at room temperature. After two PBS washes, the cells were incubated with 4 μg/ml rabbit anti-osteopontin polyclonal antibody (Abcam, Cambridge, U.K.) for 1 h. The cells were washed twice in PBS and incubated with 5 μg/ml Alexa Fluor® 568-labelled goat anti-rabbit antibody (Invitrogen, Paisley, U.K.) for 1 h. Cells were further washed with PBS and counterstained with DAPI (10 μg/ml in PBS). Coverslips were mounted onto slides using fluorescence mounting media (Dako, Denmark), viewed with a TE2000 inverted fluorescence microscope (Nikon, Surrey, U.K.) and images captured with an Orca 12-bit camera (Hamamatsu, Japan). Images were taken of the DAPI (blue) and osteopontin (red) channels using IPLab^TM^ software. Image data were quantified using MetaMorph® software. Regions of interest were created using a 70 × 70 pixel circle around the DAPI-stained nucleus by examining only the blue channel. The red fluorescence of interest was then examined within this region and the average pixel intensity of fluorescence in each region of interest assessed. 10 nuclei per treatment were evaluated.

### Detection of osteopontin by western blotting

Whole-cell lysates were prepared by scraping cells off in 1 ml ice cold PBS and pelleting by centrifugation at 1500 g for 5 min. The resultant cell pellet was resuspended in 200 μl lysis buffer. Cell extracts were incubated for 20 min at 65°C with reducing agent and sample buffer. Equal amounts of proteins (25 μg per lane) were resolved on 12% SDS-polyacrylamide gels and transferred to PVDF membranes. Membranes were blocked using 5% non-fat dried milk solution in PBS containing 0.1% (v/v) Tween-20 (PBST). Membranes were probed for 1 h with either 1 μg/ml rabbit polyclonal osteopontin (Abcam, Cambridge, U.K.) or 0.5 μg/ml mouse monoclonal β-actin (Sigma, Poole, U.K.) antibodies. Membranes were washed in PBST and then incubated with secondary 4 μg/ml goat anti-rabbit (Abcam, Cambridge, U.K.) or 1:1000 goat anti-mouse (R&D systems, Minneapolis, U.S.A.) HRP-conjugated antibodies with 1:3000 dilution *Strep*-Tactin® HRP conjugate (Bio-Rad, California, U.S.A) for a further hour with agitation. Following two washes in 5% milk-PBST and one in PBST, membranes were incubated in Immun-Star^TM^ WesternC^TM^ chemiluminescence substrate (Bio-Rad) for 5 min before detection using an XRS Gel Doc^TM^ system (Bio-Rad). Membrane images were evaluated using Quantity One® software. The molecular weights of bands were calculated against the protein standard lanes and quantified by average band density.

### Obtaining sera from human smokers

A single-centre, randomized, controlled, open-label, parallel group clinical study (approved by an Institutional Review Board) was conducted targeting enrolment of at least 120 smokers smoking their usual brand of cigarettes. U.S. adult smokers between 21 and 65 years old were enrolled in the study. Subject inclusion/exclusion criteria and baseline demographics can be found in Additional file 1: Table S1. The study was conducted in compliance with the principles of the Declaration of Helsinki. The study protocol and informed consent forms were reviewed and approved by the IntegReview Ethical Review Board (Austin, Texas, USA; http://www.integreview.com). Written, informed consent was obtained from all study subjects before participating in the study. This study was conducted according to the applicable principles of the US Code of Federal Regulations (CFR) governing the protection of human subjects (21 CFR 50), financial disclosure by clinical investigators (21 CFR 54), and IRBs (21 CFR 56).

Pre-study assessment (e.g. medical history, physical exam, hematology, serology, virology, urinalysis) showed that all subjects were generally healthy. Subjects were confirmed to smoke between 19 and 25 cigarettes per day and had been smoking for at least 3 years. Subjects resided in the clinic for approximately 8 days. Smoking status was verified during screening and daily in-clinic by measuring exhaled carbon monoxide.

On Days −2 and −1 in clinic, all subjects acclimated to smoking 20 cigarettes per day (20 CPD). The intervention started on Day 1 and consisted of maintaining or reducing to zero the number of CPD. On Day 1, subjects were randomized to 1 of 4 groups (20 CPD, 10 CPD, 5 CPD, and 0 CPD). From Day 1 until the end of the study on Day 5, subjects smoked up to the maximum allocated number of CPD/group.

Blood was collected from subjects in the 20 CPD and 0 CPD groups on Day −1 and Day +5 and serum was separated using BD diagnostics vacutainer serum separator tubes. After blood samples were drawn, tubes were inverted 5–6 times and placed upright for ≤2 h. Tubes were then centrifuged at 1300-2000 x g. Using disposable pipettes, serum was transferred into 2 ml cryovials and stored frozen at ≤ -80°C until analysis. Levels of osteopontin in sera were determined using the MSD® platform as described.

### Data analysis and statistics

Data are expressed as means ± standard deviation (S.D.). For both the *in vitro* studies and the osteopontin/IL-6 measurements in human sera, differences between treatment means were examined using Student’s unpaired *t*-tests. In the western blotting studies, data are expressed as a ratio of osteopontin to β-actin band density. Data were then normalised by dividing the band density of the treatment group by that obtained in control (DMSO-treated) cells.

## Results

### Concentration-dependent cytotoxicity of PM in endothelial cells

**Figure 1:**
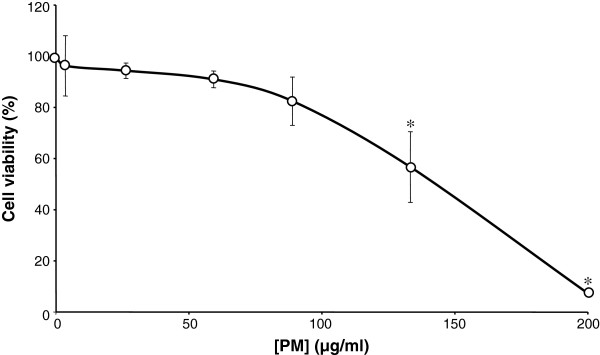
** Cytotoxicity of cigarette smoke total particulate matter (PM) in HUVECs.** Following a 24-hour exposure to the indicated concentration of PM, cell viability was assessed using neutral red uptake assays. Experiments were performed in triplicate in a single HUVEC donor, and data are representative of those obtained in 3 other donors. Data are presented as mean ± S.D. cell viability. Levels are normalised to the viability of cells not exposed to PM. *p < 0.05 compared to controls.

**Figure 2:**
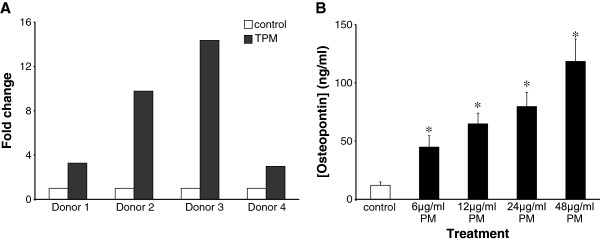
** Effects of PM treatment on osteopontin gene and protein expression. A**, HUVECs from four different donors were exposed in triplicate experiments to 88 μg/ml PM for 24 h. Gene expression was examined using a PCR array. Data are mean fold changes in gene expression normalised to DMSO controls. **B**, 24-hour exposure to PM induced the secretion of osteopontin in a concentration dependent manner. Data are mean ± S.D. osteopontin concentrations (n = 6 in each case), as detected by electrochemiluminescence. *p < 0.05 compared to controls.

To determine a non-cytotoxic range of cigarette smoke PM concentrations, neutral red viability assays were used to determine the cytotoxicity of PM exposure (0 to 200 μg/ml for 24 h) in human umbilical vein endothelial cells (HUVECs). Data from a representative donor show that between 0 and 88 μg/ml there was a modest and insignificant reduction in cell viability (p > 0.05 at each concentration; Figure 1) which reached a value of 17.5 ± 16.3% (mean ± S.D., p = 0.1) at 88 μg/ml PM. At higher concentrations, PM caused significant reductions in cell viability. Thus, a 24-h exposure to PM at concentrations of 133 and 200 μg/ml elicited 43.32 ± 23.9% (p = 0.04) and 92.7 ± 1.0% (p = 0.00001) reductions in viability, respectively (Figure 2). Given these data, PM concentrations higher than 88 μg/ml were not used in subsequent experiments.

### PM exposure induced osteopontin gene expression

To assess the effects of PM on osteopontin expression we exposed HUVECs from four different donors to 88 μg/ml PM for 24 h, after which gene expression was examined using a multi-gene PCR array. PM exposure caused osteopontin gene up-regulation of 2-fold or greater when compared to dimethylsulphoxide (DMSO) controls (Figure 2A) and this effect was consistently observed in the four donors examined. The PM-induced up-regulation was equal to or greater than the response to the positive control agent tumour necrosis factor α (10 ng/ml) in all donors tested (data not shown), indicative of a strong PM response. All subsequent studies were performed in a single donor (donor 4), chosen because other genes/proteins of interest were consistently up-regulated in this donor and it was possible to obtain more vials from this donor for additional investigations, which assists in minimising inter-individual variability.

### Effects of PM on osteopontin protein expression

To assess further the osteopontin response to PM exposure, secretion of osteopontin protein into the supernatants of PM-treated HUVECs was measured by electrochemiluminescence. As shown in Figure 2B, compared to the DMSO control, PM treatment caused a concentration-dependent increase in osteopontin secretion. Thus, media osteopontin levels following a 24-hour exposure to PM were 44.8 ± 38.3 ng/ml (p = 0.006), 64.8 ± 35.7 ng/ml (p = 0.00003), 79.7 ± 46.2 ng/ml (p = 0.00004) and 118.3 ± 71.5 ng/ml (=0.00002) in cells exposed to 6, 12, 24 and 48 μg/ml PM respectively (n = 6 at each concentration).

**Figure 3:**
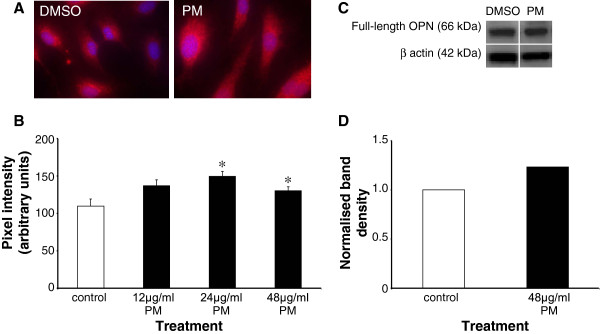
** Localisation and expression of osteopontin following PM exposure.A**, exemplar images from immunostaining studies showing the induction of osteopontin expression upon PM treatment. **B**, results of pixel intensity analysis of images such as those shown in A. Data are mean ± S.D. pixel intensities from 10 cells at each concentration. *p < 0.05 compared to controls. **C**, Western blot analysis showed the presence of osteopontin at 66 kDa, which was greater in cells treated with 48 μg/ml PM for 24 h. **D**, quantification of band densities showed a 25% increase in osteopontin levels following PM exposure.

To further elucidate the effect of PM exposure on osteopontin protein expression, experiments were conducted using immunostaining and Western blotting. The localisation and expression of cellular osteopontin protein was visualised by immunocytochemistry, using an antibody raised against amino acids 170-183 (CKSKKFRRPDIQPD) of the osteopontin peptide. When compared to controls, PM treatment caused an increase in osteopontin expression (Figure 3A). Quantification of the immunostaining by pixel intensity analysis revealed that PM at concentrations of 12, 24 and 48 μg/ml caused increases in osteopontin expression from control levels (109.7 ± 30.2 ng/ml) to 136.9 ± 25.5 ng/ml (24.7%, p = 0.09), 149.5 ± 20.9 ng/ml (36.7%, p = 0.005) and 130.2 ± 18.0 ng/ml (19%, p = 0.03) respectively (n = 10 in each case; Figure 3B). Western blotting studies further revealed that PM exposure caused an increase in total osteopontin protein compared to controls (Figure 3C). Osteopontin was detected at ~66 kDa and quantification of band densities showed that 48 μg/ml PM increased the levels of osteopontin by approximately 25% compared to the DMSO control (Figure 3D).

### A role for oxidative stress in the induction of osteopontin production by PM

**Figure 4:**
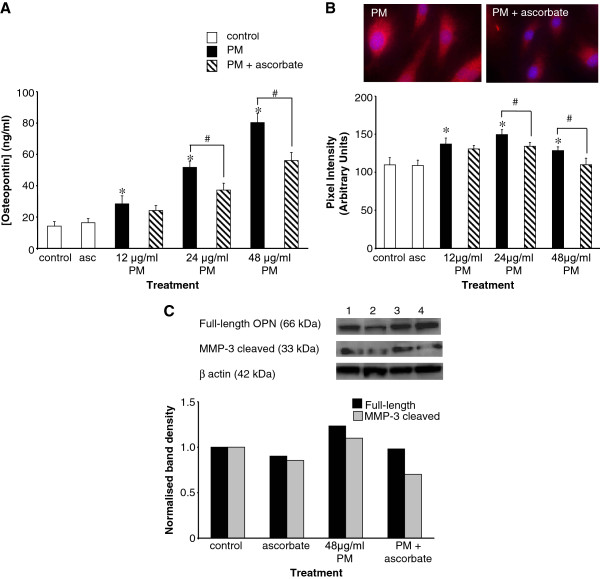
** Induction of osteopontin expression by PM is oxidative stress-dependent.A**, ascorbate pre-treatment (200 μM for 5 h) prior to a 24-hour exposure to 12, 24, or 48 μg/ml PM reduced osteopontin secretion compared to cells exposed to PM alone. Data are presented as mean ± S.D. osteopontin concentrations, as detected by electrochemiluminescence. N = 6 in each case. **B**, similar to the secretion studies, immunocytochemistry showed that ascorbate pre-treatment blunted the increase in osteopontin immunostaining caused by PM exposure. Graphical data are mean pixel intensities from 10 cells at each concentration. In A and B, *p < 0.05 compared to controls and #p < 0.05 compared to PM alone. **C**, Western blotting studies detected the presence of bands at 66 kDa and 33 kDa representing full-length and MMP-3 cleaved osteopontin, respectively. Ascorbate pre-treatment caused a reduction in the PM-induced elevation of osteopontin protein levels, which was most prominent in the MMP-3 cleaved form. Lane one, DMSO; lane two, ascorbate alone; lane three, PM; and lane four, PM with ascorbate pre-treatment. Graphical data are band densities obtained by densitometric analysis of Western blots.

Since cigarette smoke is a rich source and also a strong inducer of the cellular production of oxidants we examined the effects of pre-treatment with the antioxidant ascorbate (200 μM for 5 h) on the response to a 24-hour PM exposure. The supernatants of treated cultures were collected and osteopontin levels assessed by electrochemiluminescence. As shown in Figure 4A ascorbate significantly reduced the degree of osteopontin secretion in response to 24 and 48 μg/ml PM, causing a 28% (51.7 ± 4.1 to 37.2 ± 4.3 ng/ml, p = 0.003) and 30% (80.3 ± 6.0 to 56.0 ± 5.2 ng/ml, p = 0.004) reduction respectively (n = 6 at each concentration). Ascorbate treatment alone had no significant effect of osteopontin secretion compared to controls (n = 6, p = 0.27; Figure 4A). This effect was also observed in immunocytochemistry studies (Figure 4B) where pre-treatment of cells with ascorbate reduced the PM-induced increase in immunofluorescence, which was statistically significant at 24 and 48 μg/ml PM. Ascorbate alone had no significant effect on osteopontin expression. Western blotting studies (Figure 4C) further demonstrated that ascorbate pre-treatment reduced PM-induced osteopontin expression.

### Are MMPs involved in the PM-induced production of osteopontin?

Full-length osteopontin can be cleaved by MMP-3 to alter integrin binding and therefore cellular responses [11]. To ascertain whether this affects PM-induced osteopontin expression, Western blotting studies were performed using an antibody that detects both the full-length and the C-terminal end of the MMP-3-cleaved osteopontin fragment. Bands at ~66 kDa (full-length osteopontin) and ~33 kDa (MMP-3 cleaved) were identified following PM exposure (Figure 4C). Quantification of band densities showed that 48 μg/ml PM increased the levels of both the full-length and the MMP-3-cleaved osteopontin when compared to the DMSO controls. Pre-treatment of cells for 5 h with 200 μM ascorbate reduced the levels of both the full-length and the MMP-3-cleaved osteopontin proteins. Thus, MMP-3 cleavage may be involved in PM-induced osteopontin production.

### PM-induced expression of MMP-3 is independent of oxidative stress

**Figure 5:**
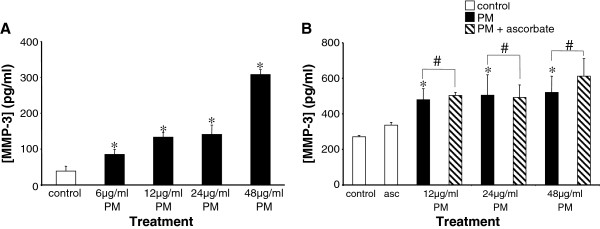
** PM-induced MMP-3 elevation occurs independently of oxidative stress. A**, PM induced a concentration-dependent increase in MMP-3 levels. Data are mean ± S.D. MMP-3 levels were assessed by electrochemiluminescence in supernatants taken from treated cells. n = 6 in each case. *p < 0.05 compared to controls. **B**, PM-induced MMP-3 expression was not affected by ascorbate pre-treatment. n = 6 in each case. *p < 0.05 compared to controls. #, p > 0.05 compared to TPM alone.

Since PM caused an increase in osteopontin expression via an oxidative stress-sensitive mechanism which may involve MMP-3 cleavage of osteopontin, we examined MMP-3 expression in HUVECs exposed to PM. Using electrochemiluminescence we constructed a concentration-response curve for the effects of PM on MMP-3 protein levels (Figure 5A). PM concentrations between 6 and 48 μg/ml PM caused significant and concentration-dependent increases in MMP-3 levels. For example, at a PM concentration of 48 μg/ml PM secreted MMP-3 levels were 307.7 ± 33.7 pg/ml, compared to 38.6 ± 33.4 pg/ml in DMSO-treated controls (p = 0.00002, n = 6). In contrast to the effects of PM on osteopontin expression, pre-incubation with ascorbate had no effect on the MMP-3 response (Figure 5B). Thus, at each concentration of PM (12, 24 and 48 μg/ml) MMP-3 protein levels in media from cells pre-treated for 5 h with 200 μM ascorbate prior to PM exposure were not significantly different from those in cells exposed to PM alone (p = 0.4, 0.7 and 0.3 respectively, in each case, n = 6).

### Osteopontin levels in sera from smokers and abstainers

Previous studies have shown that cigarette smoke promotes oxidative stress by delivering free radicals and by promoting the endogenous generation of free radicals by activated vascular cells. The oxidative stress induced by smoking is rapidly reversed on cessation [14-16]. To assess the link between smoking, oxidative stress, inflammation and osteopontin expression, we measured the levels of osteopontin protein in sera obtained from healthy smokers who were initially smoking up to 20 cigarettes per day (20 CPD) and either continued to smoke up to 20 CPD or refrained from smoking completely (0 CPD) for 5 days. In subjects in the 20 CPD group, osteopontin levels were 10626.7 ± 2333.0 pg/ml on Day -1 and 10448.8 ± 1889.1 pg/ml on Day +5 (n = 20; P = 0.68). In contrast, in those subjects in the 0 CPD group who refrained from smoking for 5 days, osteopontin levels were significantly decreased, from 10534.7 ± 2650.5 pg/ml on Day -1 to 9429.2 ± 2778.3 pg/ml on Day +5 (n = 20; p =0.03). There was also no difference between groups at Day -1 (p = 0.85). A change in the inflammation status of individuals who quit smoking was evident in the 0 CPD group since the measurement of IL-6 demonstrated that the levels of this inflammatory marker were significantly reduced from 2.25 ± 1.69 pg/ml on Day -1 to 1.89 ± 1.29 pg/ml on Day +5 (n = 20; p = 0.005).

## Discussion

The interaction between cigarette smoke, oxidative stress, inflammation and cardiovascular disease has been widely reported [14] and there is increasing evidence of a role for osteopontin as an inflammatory mediator in atherosclerosis [6,8-12]. Here, we report that exposure of endothelial cells to cigarette smoke PM *in vitro* induced the expression of osteopontin, a previously undescribed effect which may have important implications for our understanding of the association of smoking with cardiovascular disease. While a similar effect has previously been reported in human bronchial epithelial cells [17], this is the first instance where osteopontin production has been reported in response to cigarette smoke exposure in endothelial cells. Thus, the endothelium is a potential source of smoke-induced inflammatory osteopontin protein and this may play a role in cardiovascular disease. The mechanism of this endothelial response likely involves oxidative stress since osteopontin induction was diminished by treating cells with the antioxidant, ascorbate. Furthermore, our studies highlight a potential role for MMP cleavage in regulating the levels of cleaved forms of osteopontin, which have different functional attributes [11].

The analysis of gene expression was important to verify that cigarette smoke-induced changes in protein expression were due to transcriptional regulation and not transient changes in local protein concentration. This finding of altered osteopontin gene expression is in accordance with the increased osteopontin mRNA expression seen in atherosclerotic plaques *in vivo*, a measurement which closely associates with the severity of atherosclerosis and plaque calcification [13]. This suggests that the *in vitro* model used in this study to examine smoke-induced alteration of gene expression may be an appropriate one in which to further examine the inflammatory and atherogenic effects of cigarette smoke extracts.

Subsequent to the gene expression analysis we demonstrated that osteopontin protein levels were elevated following cigarette smoke extract exposure and that this effect was reduced by antioxidant pre-treatment. Cigarette smoke is both a source and a cause of the production of cellular reactive oxygen species. The pro-inflammatory effects of cigarette smoke have been well described [17-21] and these may occur through activation of oxidative stress-regulated transcription factors such as NFκB or AP-1, which regulate the expression levels of inflammatory mediators. Given our current data, we propose that endothelial-derived osteopontin may be a mediator of smoke- and oxidative stress-induced inflammation with a role in the development and/or progression of cardiovascular disease.

The functional effects of osteopontin are determined not only by its expression levels but also by its cleavage by various enzymes into functionally diverse fragments [12]. The matrix metalloproteinase family of enzymes regulate the accumulation of extracellular matrix and contribute to the growth of the atherosclerotic plaque and therefore its stability through excessive degradation of plaque cellular matrix. In particular, MMP-3 has been implicated as a strong regulator of vascular re-modelling and polymorphisms in the MMP-3 gene are linked to an increased risk of cardiac events [22]. As well as being co-localised at sites of tissue injury [12], osteopontin has been described as a substrate for MMP-3 and this may modify its integrin binding properties as well as its ability to initiate downstream signalling events [11]. In this study, we demonstrated that PM enhanced the expression of both the full-length and the MMP-3-cleaved osteopontin fragments. Interestingly, PM-induced elevation of MMP-3 itself was ascorbate-insensitive. Oxidative stress therefore may be differentially involved in the production of functionally-dissimilar osteopontin fragments and functional changes in osteopontin effects can be mediated by cigarette smoke acting on distinct and non-oxidative stress-dependent parts of the pathway.

Oxidative stress has been proposed to play a prominent role in numerous smoking-related diseases including atherosclerotic cardiovascular disease [14] and it has also been shown that the oxidative stress induced by smoking is rapidly reversible on cessation [15,16]. Given our data obtained using an *in vitro* cardiovascular disease model we hypothesised that, in a short-term study in subjects who refrained from smoking, osteopontin levels would be reduced. Our data presented here support this hypothesis by demonstrating a decrease in serum osteopontin levels in healthy subjects who quit smoking for 5 days, compared to a control group who continued to smoke 20 cigarettes per day. While further studies are needed to examine the longer-term effects of smoking cessation on both biomarkers of oxidative stress/inflammation and on the levels of osteopontin in serum, this study indicates that osteopontin can be a potential short-term biomarker of inflammation/oxidative stress.

## Limitations

In the *in vitro* work performed in this study, we examined the expression of MMP-3 protein in HUVECs. However, the expression level of MMP-3 is not the only determinant of its activity. The functional activity of the MMPs is also regulated by the tissue inhibitors of metalloproteinases (TIMPs) and the expression of TIMPs has been shown to be affected by cigarette smoke exposure [23,24]. Further studies are required to fully elucidate the role of oxidative stress on the functional activity of MMPs and its modulators, as well as on the impact of this on osteopontin expression and function, in response to cigarette smoke exposure.

In this study, we examined the levels of osteopontin protein in human sera obtained from smokers who abstained from smoking for 5 days. In this period, the levels of osteopontin in these sera were reduced compared to their levels prior to smoking abstinence. However, further studies would be required in order to understand the long-term effects of smoking cessation on osteopontin levels in serum and to assess its involvement in inflammation and its association with smoking-related cardiovascular disease.

## Conclusions

Cigarette smoke extracts induced osteopontin expression in cultured vascular endothelial cells and we propose that this may play a role in the pro-inflammatory effects of cigarette smoke. *In vitro* the induction of the osteopontin protein was oxidative stress-dependent, and serum osteopontin levels were lowered in human subjects whose oxidative stress/inflammatory burden had likely been lowered by smoking cessation. Understanding the role of the pro-inflammatory osteopontin protein in cigarette smoke-induced pathogenesis and understanding any reversible effects with smoking cessation will advance our overall understanding of cardiovascular disease development and progression.

## Competing interests

IMF and EB hold stock in British American Tobacco. EHT holds stock in R.J. Reynolds Tobacco Company.

## Authors’ contributions

EB performed all *in vitro* experimental studies and acquired all data from these studies and also measured osteopontin levels in human sera. EB also contributed to the study conception, design, analysis and interpretation of data. EHT designed, set up and monitored the clinical study from which serum samples were obtained. EHT also contributed to the analysis and interpretation of data from the clinical study. IMF contributed to the study conception, design, analysis and interpretation of data and was involved in both manuscript drafting and critical review. All authors read and approved the final manuscript.

## Authors’ information

IMF and EB are currently employees of British American Tobacco Group Research and Development. EHT is currently an employee of R. J. Reynolds Tobacco Company.

## Pre-publication history

The pre-publication history for this paper can be accessed here:

http://www.biomedcentral.com/1471-2261/12/75/prepub

## Supplementary Material

Additional file 1 **Table S1.** Subject demographics. Click here for file
