# Supplementary material for: In vitro and clinical studies examining the expression of osteopontin in cigarette smoke-exposed endothelial cells and cigarette smokers
Source: BMC Cardiovasc Disord. 2012 Sep 17;12:75. doi: 10.1186/1471-2261-12-75 (PMC3465212; doi:10.1186/1471-2261-12-75)
Supplement: Additional file 1 — Table S1. Subject demographics. [file 1471-2261-12-75-S1.docx]

**Supplementary Information**

**Subject inclusion and exclusion criteria**

**Target Population**

The study target population will include male and female, verified adult smokers who are considered generally healthy, as determined by the Investigator.

**Inclusion Criteria**

Eligible subjects will meet all the following criteria to be enrolled in this study:

1. Are able to comprehend and willing to sign an informed consent form (ICF).

2. Are male or female adult smokers, 21 to 65 years old.

3. Self-report smoking 19 to 25 CPD for ≥ 3 years prior to screening (at the prescreening interview and Visit 1).

4. Have smoked the same UB cigarette for the last 3 months prior to screening.

5. Have clinic-verified CPD use of 19 to 25 CPD (at Visit 2). Verification occurs by counting and averaging CPDs from 3-day pre-clinic smoked filter collections.

6. Have exhaled carbon monoxide (ECO) ≥ 15 parts per million (ppm).

7. Are generally healthy based on screening assessments.

8. Are willing and able to adhere to study procedures, including willingness to reduce or abstain from smoking for approximately 5.5 days if randomized to a “Reducer” or the “Abstainer” group.

9. Are able to read, understand, and complete questionnaires in English.

**Exclusion Criteria**

Subjects who meet any of the following criteria will be excluded from the study:

1. Smoke cigarettes that have no filter or have charcoal filters, or use any non-tobacco

burning cigarettes including tobacco-heating cigarettes or electronic cigarettes.

2. Use any type of tobacco-based products (e.g., smokeless tobacco) other than

conventional cigarettes within 3 months preceding Confinement.

3. Have smoked a cigar within 2 months of clinic confinement.

4. Have negative result in NicCheck I (urine cotinine) screen.

5. Intend to quit smoking leading up to study participation (defined as planning a quit attempt within 1 month of Visit 1).

6. Are pregnant (positive urine test), lactating (self-reported), or intend to become pregnant during the study.

7. Have clinically significant or unstable acute or chronic medical conditions at screening, as judged by the Investigator (e.g., based on screening assessments such as medical history, physical examination, electrocardiogram [ECG], and laboratory values).

8. Have a positive serology test result (hepatitis A, B, or C, or human immunodeficiency virus [HIV]) at screening.

9. Test positive for drugs of abuse (positive test result due to prescribed medications may be considered acceptable, at the discretion of the Investigator).

10. Test positive for breath alcohol (by breathalyzer) (positive test result due to prescribed medications may be considered acceptable, at the discretion of the Investigator).

11. Have a body mass index (BMI) > 50 kg/m2.

12. Have participated in a clinical study and/or received an investigational product within 1 month prior to screening.

13. History of alcohol abuse or illicit drug use ≤ 12 months before Confinement. NOTE: Subjects who self-report drinking more than 14 servings of alcoholic beverages per week (1 serving = 12 ounces of beer, 6 ounces of wine, or 1 ounce of liquor) will be excluded.

14. Are females or males who have used a nicotine-replacement therapy within 3 months prior to clinic Confinement, use blood pressure medication, or use cholesterol medication, or are females > 35 years of age who use oral contraceptives or hormone-replacement therapies.

15. Have donated or received blood products ≤ 1 month before Confinement.

16. Are employed (or have been employed within the last 3 months) by the tobacco industry, the Investigator, or the study center, or are family members of the study center employees or Investigator.

17. Are subjects who, for any reason, are deemed by the Investigator to be inappropriate for this study, including subjects who are unable to communicate or are unwilling to cooperate with the Investigator, or excess subjects (if enrollment has been met for the group).

**Table 1. Subject demographics.**

|  | **Maintainers**  **20 CPD** | **Reducers**  **10 CPD** | **Reducers**  **5 CPD** | **Abstainers**  **0 CPD** |
| --- | --- | --- | --- | --- |
| **n** | 30 | 30 | 32 | 31 |
|  |  |  |  |  |
| **Gender** |  |  |  |  |
| Female | 12 (40%) | 12 (40%) | 13 (41%) | 13 (42%) |
| Male | 18 (60%) | 18 (60%) | 19 (59%) | 18 (58%) |
|  |  |  |  |  |
| **Age (years)** |  |  |  |  |
| Mean (SD) | 38.4 (11.49) | 44.0 (11.18) | 42.3 (11.36) | 42.8 (9.02) |
| Median | 39 | 45.5 | 44.5 | 43 |
| Min, Max | 21, 59 | 22, 63 | 21, 59 | 21, 60 |
|  |  |  |  |  |
| **Ethnicity** |  |  |  |  |
| Hispanic or Latino | 5 (17%) | 3 (10%) | 5 (16%) | 5 (16%) |
| Not Hispanic or Latino | 25 (83%) | 27 (90%) | 27 (84%) | 26 (84%) |
|  |  |  |  |  |
| **Race** [1] |  |  |  |  |
| White | 25 (83%) | 27 (90%) | 24 (75%) | 24 (77%) |
| Black or African American | 5 (17%) | 4 (13%) | 9 (28%) | 7 (23%) |
| Asian | 0 | 0 | 0 | 0 |
| Native Hawaiian or Other Pacific Islander | 0 | 0 | 0 | 0 |
| American Indian or Alaska Native | 0 | 1 (3%) | 0 | 0 |
|  |  |  |  |  |
| **Height (cm)** |  |  |  |  |
| Mean (SD) | 170.0 (8.1) | 172.3 (11.1) | 172.0 (9.1) | 172.9 (9.9) |
| Median | 170.0 | 173.0 | 171.8 | 175.0 |
| Min, Max | 153.0, 186.0 | 151.0, 190.0 | 156.0, 186.7 | 153.5, 192.5 |
|  |  |  |  |  |
| **Weight (kg)** |  |  |  |  |
| Mean (SD) | 79.8 (17.8) | 80.9 (19.4) | 87.5 (19.7) | 84.1 (16.6) |
| Median | 79.3 | 82.8 | 86.4 | 81.0 |
| Min, Max | 48.6, 110.4 | 49.2, 122.0 | 44.5, 125.6 | 46.4, 131.0 |
|  |  |  |  |  |
| **Body Mass Index (BMI; kg/m^2^)** |  |  |  |  |
| Mean (SD) | 27.4 (5.3) | 27.0 (4.6) | 29.6 (6.6) | 28.1 (5.1) |
| Median | 26.6 | 26.1 | 28.0 | 27.1 |
| Min, Max | 19.1, 36.9 | 20.5, 37.0 | 16.1, 43.6 | 19.7, 46.1 |
